# Simultaneous determination of 3-hydroxypropionic acid, methylmalonic acid and methylcitric acid in dried blood spots: Second-tier LC-MS/MS assay for newborn screening of propionic acidemia, methylmalonic acidemias and combined remethylation disorders

**DOI:** 10.1371/journal.pone.0184897

**Published:** 2017-09-15

**Authors:** Péter Monostori, Glynis Klinke, Sylvia Richter, Ákos Baráth, Ralph Fingerhut, Matthias R. Baumgartner, Stefan Kölker, Georg F. Hoffmann, Gwendolyn Gramer, Jürgen G. Okun

**Affiliations:** 1 Department of General Pediatrics, Division of Neuropediatrics and Metabolic Medicine, Center for Pediatric and Adolescent Medicine, University Hospital Heidelberg, Heidelberg, Germany; 2 Department of Pediatrics, University of Szeged, Szeged, Hungary; 3 Children’s Research Center, Division of Metabolism, University Children’s Hospital Zurich, Zurich, Switzerland; Indian Institute of Chemical Technology, INDIA

## Abstract

**Background and aims:**

Increased propionylcarnitine levels in newborn screening are indicative for a group of potentially severe disorders including propionic acidemia (PA), methylmalonic acidemias and combined remethylation disorders (MMACBL). This alteration is relatively non-specific, resulting in the necessity of confirmation and differential diagnosis in subsequent tests. Thus, we aimed to develop a multiplex approach for concurrent determination of 3-hydroxypropionic acid, methylmalonic acid and methylcitric acid from the same dried blood spot (DBS) as in primary screening (second-tier test). We also set out to validate the method using newborn and follow-up samples of patients with confirmed PA or MMACBL.

**Methods:**

The assay was developed using liquid chromatography–tandem mass spectrometry and clinically validated with retrospective analysis of DBS samples from PA or MMACBL patients.

**Results:**

Reliable determination of all three analytes in DBSs was achieved following simple and fast (<20 min) sample preparation without laborious derivatization or any additional pipetting steps. The method clearly distinguished the pathological and normal samples and differentiated between PA and MMACBL in all stored newborn specimens. Methylcitric acid was elevated in all PA samples; 3-hydroxypropionic acid was also high in most cases. Methylmalonic acid was increased in all MMACBL specimens; mostly together with methylcitric acid.

**Conclusions:**

A liquid chromatography–tandem mass spectrometry assay allowing simultaneous determination of the biomarkers 3-hydroxypropionic acid, methylmalonic acid and methylcitric acid in DBSs has been developed. The assay can use the same specimen as in primary screening (second-tier test) which may reduce the need for repeated blood sampling. The presented preliminary findings suggest that this method can reliably differentiate patients with PA and MMACBL in newborn screening. The validated assay is being evaluated prospectively in a pilot project for extension of the German newborn screening panel (‟Newborn screening 2020”; Newborn Screening Center, University Hospital Heidelberg).

## Introduction

**Fig 1:**
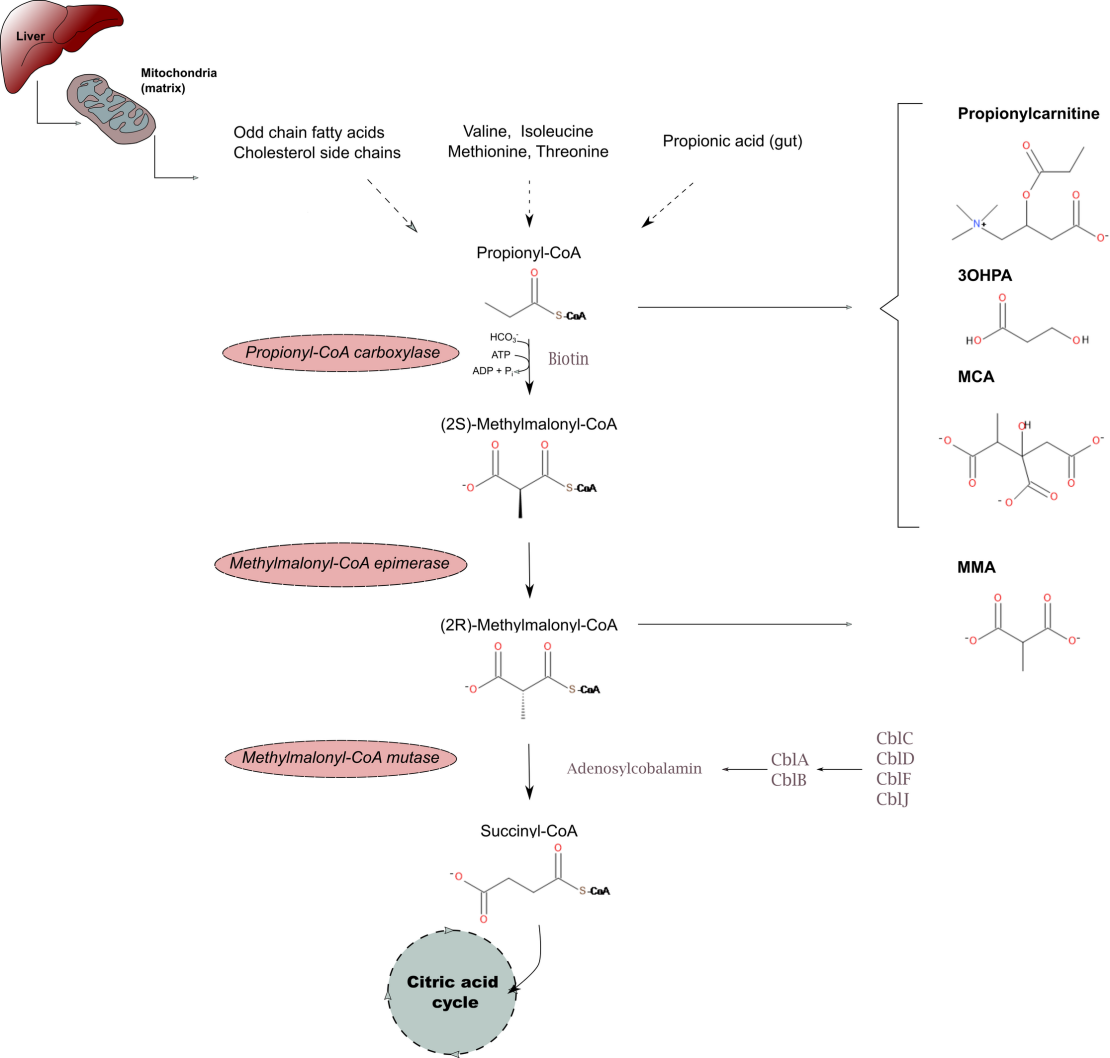
Biochemical pathway of propionate metabolism (simplified). 3OHPA: 3-hydroxypropionic acid; MMA: methylmalonic acid; MCA: methylcitric acid; Cbl: cobalamin. Drawn using Inkscape 0.91pre4 (Open Source Software licensed under the GNU General Public License).

Propionic acidemia (PA; OMIM 606054) and methylmalonic acidemias including methylmalonyl-CoA mutase, methylmalonyl-CoA epimerase and cobalamin (Cbl) A, B, C, D, F and J deficiencies (MMACBL; OMIM 251000, 251120, 251100, 251110, 277400, 277410, 277380 and 614857, respectively) are rare but potentially severe autosomal recessive disorders of propionate catabolism (Fig 1) [1–6]. Methylmalonyl-CoA mutase deficiency can be complete (MMAmut(0)) or partial (MMAmut(-)) [1]. The clinical symptoms of PA and MMACBL are widely variable and, in many cases, potentially life-threatening [1, 5, 6]. Various organs including the nervous and gastrointestinal systems, the heart and the kidney can be affected [1, 5, 6]. Accordingly, an early diagnosis by means of newborn screening (NBS) has been recommended by the American College of Medical Genetics and National Academy of Clinical Biochemistry [2–4] for PA, MMAmut deficiency and Cbl A and B defects, disorders having the highest potential for severe outcome [1, 5, 6].

In NBS, an increased propionylcarnitine (C3) level in dried blood spots (DBSs) is suggestive for either PA or MMACBL [1]. However, C3 elevation is not specific for these disorders due to an overlap of its concentration in affected and unaffected newborns, particularly in premature infants, which results in high false-positive rates [1, 7]. The use of analyte ratios such as C3/acetylcarnitine (C3/C2) improves specificity, but still cannot differentiate between PA and MMACBL [1, 8]. This necessitates confirmation of primary screening results and differential diagnosis using more specific assays [1].

Traditionally, confirmation of PA has been performed from urine by detecting elevated levels of various biomarkers including 3-hydroxypropionic acid (3OHPA) and methylcitric acid (MCA), together with normal methylmalonic acid (MMA) concentrations (Fig 1) [1]. In MMACBL, urinary MMA and MCA are increased; 3OHPA may also be elevated (Fig 1) [1]. However, urine can only be obtained via patient recall which markedly delays time to diagnosis and initiation of treatment and may also generate unnecessary parental stress [1]. In turn, methods using the same DBS specimen as in the primary screening (second-tier tests) may reportedly reduce time to diagnosis and the number of recalls [9]. However, previous second-tier assays for PA and MMACBL [10–17] did not allow concurrent measurement of these biomarkers from DBS and generally utilized time-consuming and laborious sample preparation [10–14].

Importantly, a simultaneous and selective measurement of all three relevant diagnostic biomarkers from DBS could allow the evaluation of their individual diagnostic values in DBS for differentiation between PA and MMACBL and as a result could reinforce the analytical confidence of the diagnosis. In addition, the applied method must also be able to separate two additional interfering substances, lactic acid and succinic acid (isobaric with 3OHPA and MMA, respectively), being highly abundant in blood [1].

Therefore, our aim was to develop a liquid chromatography–tandem mass spectrometry (LC-MS/MS) assay for the simultaneous determination of the biomarkers 3OHPA, MMA and MCA from DBS, while separating the isobaric metabolites lactic acid and succinic acid. We also set out to keep sample preparation simple and fast for easier inclusion of the assay in the routine workflow. To assess the relevance of the three biomarkers in DBS, we retrospectively analyzed specimens from known patients with PA or MMACBL before and during therapy using the successfully developed assay.

## Materials and methods

### Reagents

Unlabelled 3OHPA, lactic acid, succinic acid, MMA, MCA and zinc sulfate heptahydrate (ZnSO_4_ · 7H_2_O) were purchased from Sigma-Aldrich (St. Louis, MO, USA); deuterated internal standards (ISs) d_3_-MMA from Cambridge Isotope Laboratories (Andover, MA, USA); d_3_-lactic acid and d_3_-MCA from CDN Isotopes (Pointe-Claire, Quebec, Canada); methanol, acetonitrile and formic acid (all ULC-MS grade) from Biosolve (Valkenswaard, The Netherlands). Ultrapure water (18.2 MΩ.cm), filtered through a 0.22 μm pore size membrane, was obtained from a Thermo Barnstead GenPure Pro UV-TOC/UF system (Thermo Fisher Scientific, Waltham, MA, USA).

### Sample preparation

Two spots 3.2 mm in diameter of each of the DBS calibrators, Quality Controls (QCs) and the patient samples (corresponding to 6.4 μl blood) were punched out from filter cards and placed into Merck Ultrafree-MC VV 0.1 µm centrifugal filter units (Darmstadt, Germany). The IS working solution (composition: 20, 1.7, and 0.50 µM for d_3_-lactic acid, d_3_-MMA and d_3_-MCA, respectively) was prepared fresh daily by a 5-fold dilution of a deuterated IS intermediate stock solution (stored in aliquots at -20°C) with methanol/water 50/50 (v/v). To each sample, 120 μl IS working solution plus 10 μl 50 mM ZnSO_4_ solution (in methanol) were added. The samples were sealed, vortexed for 5 min, and centrifuged for 10 min at 5000 × *g* in a Heraeus Biofuge Pico centrifuge (Hanau, Germany) at ambient temperature. After removal of the lid and filter, the tubes were placed directly into the autosampler. The analyte levels in DBS calibrators Cal0 to Cal6 (stored at -20°C) were as follows: 0, 12.5, 25, 50, 100, 200, 400 μM for 3OHPA; 0, 1.25, 2.5, 5.0, 10, 20, 40 μM for MMA; and 0, 0.25, 0.50, 1.0, 2.0, 4.0, 8.0 μM for MCA, respectively. The analyte levels in QC1, QC2 and QC3 (stored at -20°C) were the following: 20, 60, 100 μM for 3OHPA; 2.0, 30, 100 μM for MMA; and 0.40, 1.2, 4.0 μM for MCA, respectively. The preparation of standard stock solutions, DBS calibrators and QCs are further detailed in S1 Text.

### LC-MS/MS instruments and settings

A Waters Acquity I-Class UPLC system (Binary Solvent Manager, thermostatic Column Manager and FTN Sample Manager) and a Waters TQ-S triple quadrupole MS/MS were used, controlled by MassLynx 4.1 software (all Waters, Milford, MA, USA). MS/MS data were evaluated with TargetLynx 4.1 software (Waters, Milford, MA, USA).

The chromatographic separation of the analytes was performed on a Phenomenex Gemini C6-Phenyl 150×2.0 mm, 3 μm analytical column and a SecurityGuard C6-Phenyl 20 mm I.D. cartridge guard column (both Phenomenex, Torrance, CA, USA). Eluent A consisted of ultrapure water plus 0.4% formic acid (ULC-MS grade). Eluent B consisted of methanol/acetonitrile 50/50 (both ULC-MS grade). Both eluents were prepared weekly. The column was tempered to 25°C. The autosampler settings were the following: injection volume: 5 μl; sample compartment temperature: 5°C. Gradient elution at a flow of 200 μl/min was performed by changing %B as follows: 0.0-1.5 min: 10%; 1.5-2.5 min: 10% to 30%; 2.5-4.0 min: 30%; 4.0-4.1 min: 30% to 10%; 4.1-10.0 min: 10%. Experience with further extraction solvents, columns and eluents, derivatization and isocratic elution obtained during method development are provided in S1 Table.

MS/MS settings were optimized by means of consecutive syringe infusions of 10 μM solutions for each analyte and IS, dissolved in ultrapure water. All analytes and ISs were measured in negative electrospray ion mode; the dwell times were 20 ms each. Optimized MS/MS settings are summarized in Table 1. The final ion source settings were the following: desolvation gas flow = 800 L/h; cone gas flow = 150 L/h; nebulizer = 6.0 bar; capillary voltage = 2.5 kV; desolvation temperature = 650°C; source temperature = 150°C.

**Table 1:** Optimized MS/MS settings in the second-tier LC-MS/MS assay (negative electrospray ion mode).

Analyte	Parent mass (Da)	Daughter mass (Da)	Cone voltage (V)	Collision voltage (V)
**3OHPA**	88.9	59.0	-48	-10
**Lactic acid**	88.9	43.0	-46	-11
**d_3_-Lactic acid**	92.0	45.1	-46	-11
**Succinic acid**	116.9	72.9	-47	-12
**MMA**	117.0	73.0	-30	-12
**d_3_-MMA**	120.0	76.1	-30	-10
**MCA**	205.0	125.1	-35	-13
**d_3_-MCA**	208.0	87.1	-35	-18

3OHPA: 3-hydroxypropionic acid; MMA: methylmalonic acid; MCA: methylcitric acid.

### Application of the LC-MS/MS test on patient samples with PA and MMACBL and external quality control samples

Clinical validation of the LC-MS/MS assay was performed retrospectively using 79 DBS samples from 32 different patients with PA or MMACBL from the NBS and Metabolic Centers in Heidelberg (Germany), Szeged (Hungary) and Zurich (Switzerland). In 14 patients, the newborn specimens prior to therapy initiation were also available (PA: *n* = 4; CblB *n* = 1; CblC *n* = 9); all other samples were obtained during therapy. Storage duration of the patient specimens before second-tier analysis in median (range) was 0.61 (0.01-8.90) years. For additional characterization of samples, we used the first–tier acylcarnitine profiles (electrospray ionization-MS/MS [18–20]) determined both at time of blood sampling at the respective site providing the specimen, as well as after storage (measured at the NBS Centers Heidelberg and Szeged). First–tier cutoff values for C3 and C3/C2 in the respective laboratories were the following: Heidelberg 5.5 μM and 0.22; Szeged: 5.75 μM and 0.26; and Zurich: 6.0 μM and 0.17. The 3OHPA, MMA and MCA levels were also measured in External Quality Assurance DBS samples for NBS (*n* = 7) from ERNDIM.

The validated assay is currently being evaluated prospectively in a pilot project for the extension of the German NBS panel named ‟Newborn screening 2020” at the NBS Center Heidelberg. In that prospective study, the reported LC-MS/MS assay is being applied as second-tier test using the same DBS specimen as in the primary screening.

All procedures followed were in accordance with the ethical standards of the Helsinki Declaration of 1975, as revised in 2000. Written informed consent for regular newborn screening had been obtained from the parents of all individuals at time of blood sampling if required by national legislation. The anonymized, retrospective re-analysis of the specimens in the present study was approved by the Ethical Committees of the University of Szeged (217/2016-SZTE) and the University Children’s Hospital Zurich (2014-0211). At the University Hospital Heidelberg, parents of patients under treatment gave written informed consent for sample analysis for establishment of the second-tier strategies for the study NBS 2020. The study NBS 2020 was approved by the Ethical Committee of the University of Heidelberg (S-533/2015).

## Results

### Method validation

The linearity, the limit of detection (LOD) and the lower limit of quantitation (LLOQ) were tested with various concentrations of calibrator DBSs (*n* = 10). LOD was defined as S/N = 3; LLOQ was defined as the lowest concentration measured with a coefficient of variation (CV) lower than 20%. The coefficient of determination for linearity (R^2^) was 0.9702 for 3OHPA, 0.9946 for MMA and 0.9926 for MCA (Table 2). The LOD and LLOQ values for 3OHPA, MMA and MCA were: 15 μM, 1.5 μM, 0.05 μM; and 20 μM, 2.5 μM, 0.07 μM, respectively (Table 2).

**Table 2:** Assay validation in calibrator dried blood spots (*n* = 10).

Analyte	Linearity (R^2^)	LOD (μM)	LLOQ (μM)	Retention time (min)
**3OHPA**	0.9702	15	20	2.4±0.01
**MMA**	0.9946	1.5	2.5	3.9±0.01
**MCA**	0.9926	0.05	0.07	3.7±0.01 and 4.2±0.01

3OHPA: 3-hydroxypropionic acid; MMA: methylmalonic acid; MCA: methylcitric acid; R^2^: coefficient of determination; LOD: limit of detection; LLOQ: lower limit of quantitation.

**Fig 2:**
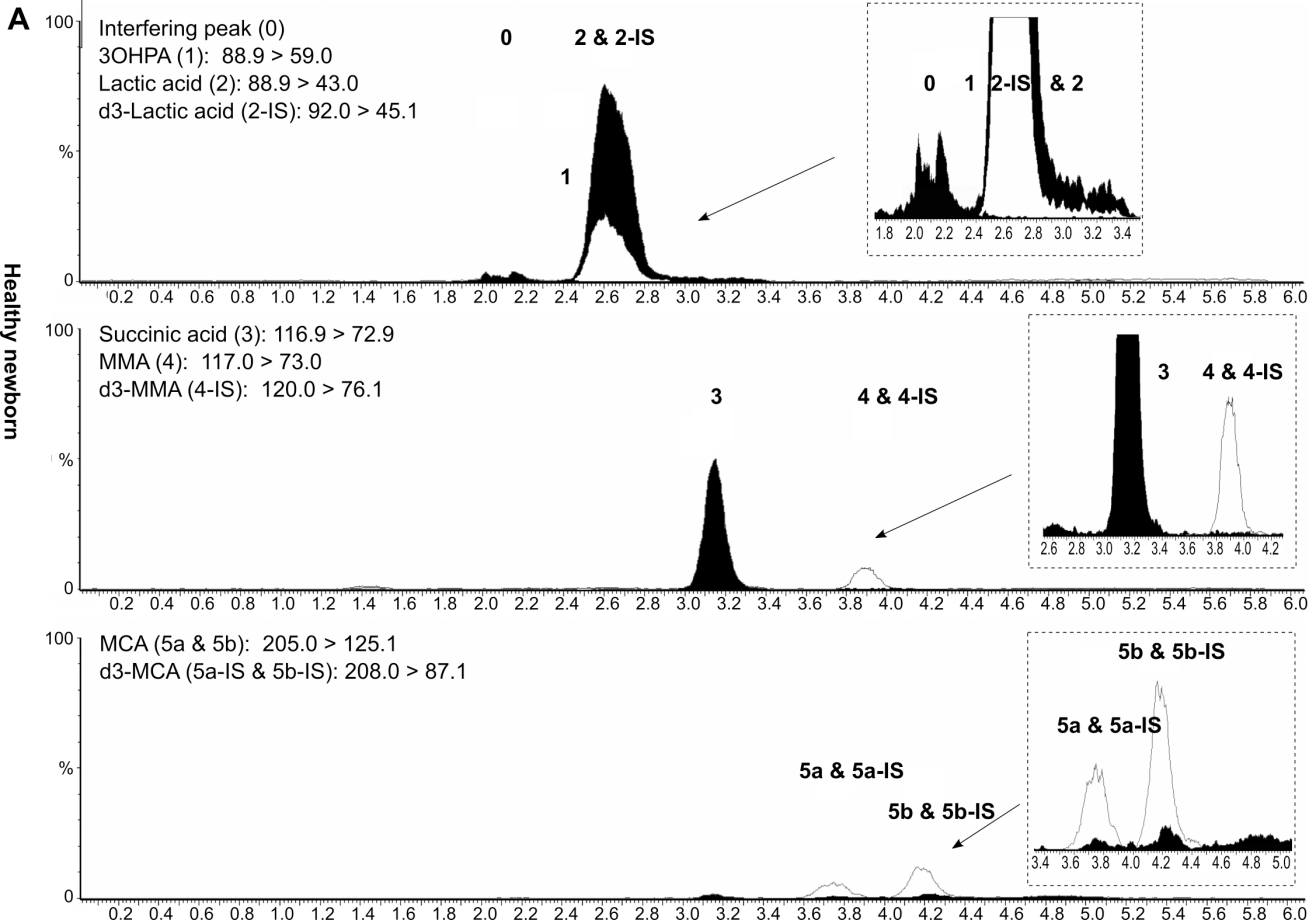
LC-MS/MS chromatogram of DBS from a healthy newborn. Peak 1: 3-hydroxypropionic acid (3OHPA); peak 2: lactic acid; peak 3: succinic acid; peak 4: methylmalonic acid (MMA); peaks 5a and 5b: methylcitric acid (MCA); peak 0: interfering peak; DBS: dried blood spot. Numbered black peaks: unlabelled analytes; numbered unfilled peaks: deuterated internal standards. All intensities (Y-axes) have been normalized to 4*10^5^ cps for better comparability (not on the inserted zoom-in figures) and have been plotted against retention time (X-axes). Chromatograms were generated by MassLynx 4.1 (Waters, Milford, MA, USA) and modified using Inkscape 0.91pre4 (Open Source Software licensed under the GNU General Public License).

**Fig 3:**
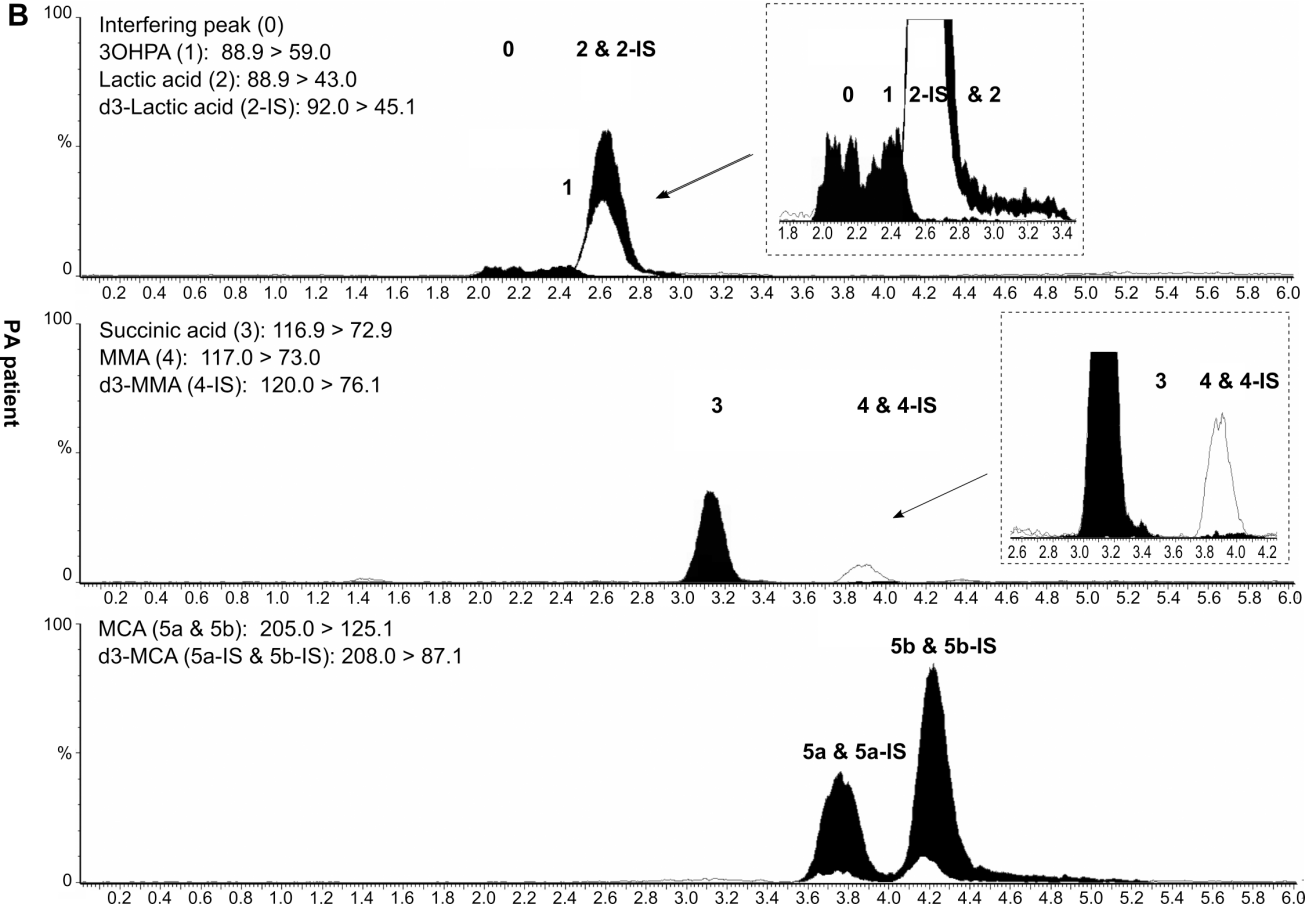
LC-MS/MS chromatogram of DBS from a PA patient. Peak 1: 3-hydroxypropionic acid (3OHPA); peak 2: lactic acid; peak 3: succinic acid; peak 4: methylmalonic acid (MMA); peaks 5a and 5b: methylcitric acid (MCA); peak 0: interfering peak; DBS: dried blood spot; PA: propionic acidemia. Numbered black peaks: unlabelled analytes; numbered unfilled peaks: deuterated internal standards. All intensities (Y-axes) have been normalized to 4*10^5^ cps for better comparability (not on the inserted zoom-in figures) and have been plotted against retention time (X-axes). Chromatograms were generated by MassLynx 4.1 (Waters, Milford, MA, USA) and modified using Inkscape 0.91pre4 (Open Source Software licensed under the GNU General Public License).

**Fig 4:**
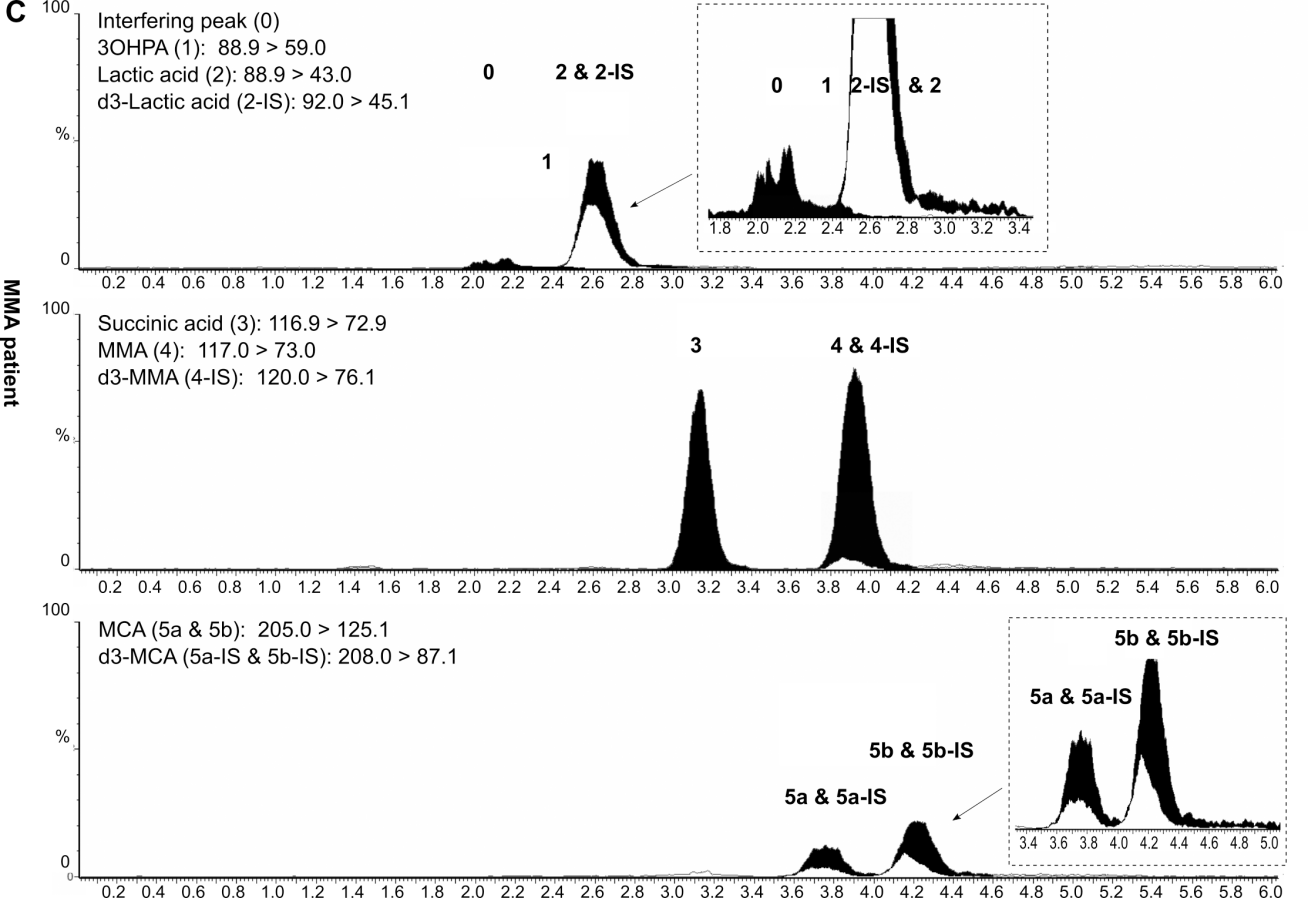
LC-MS/MS chromatogram of DBS from a MMAmut(0) patient. Peak 1: 3-hydroxypropionic acid (3OHPA); peak 2: lactic acid; peak 3: succinic acid; peak 4: methylmalonic acid (MMA); peaks 5a and 5b: methylcitric acid (MCA); peak 0: interfering peak; DBS: dried blood spot; MMAmut(0): methylmalonyl-CoA mutase deficiency (complete). Numbered black peaks: unlabelled analytes; numbered unfilled peaks: deuterated internal standards. All intensities (Y-axes) have been normalized to 4*10^5^ cps for better comparability (not on the inserted zoom-in figures) and have been plotted against retention time (X-axes). Chromatograms were generated by MassLynx 4.1 (Waters, Milford, MA, USA) and modified using Inkscape 0.91pre4 (Open Source Software licensed under the GNU General Public License).

The retention times of 3OHPA and MMA were 2.4 min and 3.9 min, respectively. Of note, MCA and d_3_-MCA are both detected in two chromatographic peaks (Figs 2–4). In case of d_3_-MCA, the first peak at 3.7 min reportedly corresponds to the (2R, 3S) and (2S, 3R) stereoisomers; and the second peak at 4.2 min to (2R, 3R) and (2S, 3S) [21, 22]. Of the four possible stereoisomers of unlabeled MCA, only two, (2R, 3S) and (2S, 3S) are physiological [21, 22]. The respective MCA and d_3_-MCA peaks were summed during the evaluation of the concentrations.

The resolution of critical isobaric analyte pairs was determined using the Cal5 DBS calibrator (*n* = 10), by averaging retention times (*t*_*1*_ and *t*_*2*_) and peak widths at half peak height (*w*_*0*.*5*,*1*_ and *w*_*0*.*5*,*2*_) in 10 injections. Resolution (*R*_*s*_) was calculated via the following equation: *R*_*s*_ *= 1*.*18(t*_*2*_*-t*_*1*_*)/(w*_*0*.*5*,*1*_*+w*_*0*.*5*,*2*_*)*. The critical isobaric pair succinic acid and MMA were baseline resolved (*R*_*s*_ = 2.7). As the isobaric metabolites 3OHPA and lactic acid had specific daughter ions and therefore specific MRM transitions (Table 1), a chromatographic separation was not necessary for their determination. Despite extensive efforts, however, the 3OHPA peak could not be fully chromatographically separated from an interference peak having identical MRM transitions (*R*_*s*_<1.0) (peaks 0 and 1 on Figs 2–4).

Interday and intraday precisions, variations between multiple injections of the same sample extract and recoveries were examined in QCs at three different concentrations (*n* = 10) (Table 3). With the exception of the lowest MMA level (2.0 μM), the CV values were lower than 15% (Table 3). The recoveries ranged from 99.0-109.4%, except for the lowest 3OHPA level (20 μM) (Table 3).

**Table 3:** Assay validation in dried blood spots using quality control samples at three concentrations (*n* = 10).

Analyte	Analyte level (μM)	CV (%) interday	CV (%) intraday	CV (%) between injections	Recovery (%)
**3OHPA**	**20**	9.2	10.8	10.7	136.8
	**60**	14.3	13.4	8.0	99.2
	**100**	6.2	10.6	7.9	106.8
**MMA**	**2.0**	32.9	20.2	10.6	108.1
	**30**	9.3	9.1	5.0	99.0
	**100**	12.9	10.5	3.3	105.0
**MCA**	**0.40**	10.2	9.1	2.2	102.3
	**1.2**	6.9	6.5	2.7	104.4
	**4.0**	11.3	6.0	2.0	109.4

3OHPA: 3-hydroxypropionic acid; MMA: methylmalonic acid; MCA: methylcitric acid; CV: coefficient of variation.

### Testing of the present assay with clinical specimens

For the determination of normal biomarker concentrations in the second-tier test, we used residual DBS samples (*n* = 250) after primary NBS for amino acids and acylcarnitines. Blood samples (collected between 36-72 hours of age) with C3 levels lower than or equal to 4.5 μM and ratios C3/C2 lower than or equal to 0.18 were randomly selected and anonymized prior to the analysis. The 99.9^th^ percentiles in DBS of these newborns, chosen as cutoff values for the second-tier test, were: 3OHPA: 38.13 μM; MMA: 2.29 μM; and MCA: 0.08 μM.

Results of the clinical validation of the LC-MS/MS assay by means of retrospective analysis of stored patient samples are summarized in Table 4. The second-tier test clearly distinguished the pathological and normal samples and differentiated between PA and MMACBL in all newborn specimens, despite marked degradation of C3 after storage (up to 95%) [23, 24], as compared with C3 levels at time of blood sampling. Similarly, most specimens obtained during therapy showed biomarker patterns characteristic for the respective disorder. As concerns PA samples, 3OHPA was normal in 2 out of 4 untreated newborn PA specimens and in 7 out of 21 PA samples on therapy. In these cases, only the elevated MCA could confirm the underlying disease. It has to be considered, that all four newborn PA specimens were subject to long-term storage (range 3.45-8.90 years). Storage time before analysis in the two NBS PA samples with normal 3OHPA was especially long (8.86 and 8.90 years).

**Table 4:** Results of dried blood spot samples from patients with known diagnosis: Retrospective analysis of NBS specimens (untreated) and samples during therapy. Data are provided as medians (ranges).

		First-tier test (initial analysis shortly after blood sampling)		Second-tier test		
	Biomarker	C3 (μM)	C3/C2	3OHPA (μM)	MMA (μM)	MCA (μM)
	Cutoff	5.8 (5.5-6.0)	0.22 (0.17-0.26)	38.13	2.29	0.08
**NBS**	**Controls (*n* = 250)**	1.9 (0.7-4.3)	0.08 (0.04-0.18)	n.d. (n.d.-38.8)	0.50 (n.d.-2.3)	0.02 (n.d.-0.08)
**NBS**	**PA (*n* = 4)**	14.7 (10.4-25.0)	0.51 (0.24-1.94)	153.2 (20.9-367.2)	1.8 (n.d.-2.0)	2.06 (0.11-2.85)
**NBS**	**CblB (*n* = 1)**	6.3	0.31	42.1	21.9	0.04
**NBS**	**CblC (*n* = 9)**	7.1 (2.9-10.1)	0.39 (0.24-2.26)	33.2 (n.d.-117.3)	28.9 (5.1-640.3)	0.29 (0.04-1.40)
**Treated**	**PA (*n* = 21)**	24.9 (3.2-80.7)	1.12 (0.23-4.31)	78.7 (n.d.-775.2)	0.1 (n.d.-1.6)	1.72 (0.11-7.00)
**Treated**	**MMAmut(0) (*n* = 4)**	39.2 (5.0-48.4)	1.04 (0.45-1.24)	86.6 (31.6-136.3)	1727.6 (846.3-3464.4)	3.65 (0.75-8.85)
**Treated**	**MMAmut(-) (*n* = 11)**	7.3 (3.4-12.0)	0.43 (0.20-0.57)	n.d. (n.d.-41.8)	22.8 (4.6-49.6)	0.08 (0.02-0.15)
**Treated**	**CblA (*n* = 2)**	5.9 (5.2-6.6)	0.41 (0.30-0.52)	27.9 (21.9-33.9)	70.0 (10.8-129.2)	0.28 (0.16-0.39)
**Treated**	**CblB (*n* = 17)**	15.6 (1.3-23.3)	0.88 (0.08-1.44)	40.4 (n.d.-68.8)	193.2 (n.d.-460.8)	0.32 (n.d.-0.55)
**Treated**	**CblC (*n* = 10)**	4.3 (1.1-8.8)	0.37 (0.08-1.62)	24.7 (n.d.-43.1)	9.5 (n.d.-78.0)	0.11 (n.d.-0.47)

NBS: newborn screening; PA: propionic acidemia; Cbl: cobalamin; MMAmut: methylmalonyl-CoA mutase deficiency; C3: propionylcarnitine; C2: acetylcarnitine; 3OHPA: 3-hydroxypropionic acid; MMA: methylmalonic acid; MCA: methylcitric acid; n.d.: not detectable.

Of note, in four MMACBL cases under treatment (MMAmut(-) *n* = 1; CblC *n* = 3), the second-tier results were still indicative of the disorder when the initial first-tier C3 and C3/C2 values were already in the normal range. In five additional patients under therapy (CblB *n* = 3; CblC *n* = 2), the first-tier and second-tier results were both normal. Representative LC-MS/MS chromatograms of DBS specimens from a healthy newborn, a PA and a MMAmut(0) patient are presented in Figs 2–4, respectively.

### Testing of the present assay with external quality control samples

The 3OHPA, MMA and MCA levels were measured in External Quality Assurance DBS samples for NBS (*n* = 7) from ERNDIM. The second-tier test clearly differentiated between PA and MMACBL in all External Quality Assurance specimens (Table 5).

**Table 5:** Results of External Quality Assurance dried blood spot samples (*n* = 7).

	Second-tier test			Diagnosis according to second-tier test result
Biomarker	3OHPA (μM)	MMA (μM)	MCA (μM)	
Cutoff	38.13	2.29	0.08	
**ERNDIM No. 1**	90.33	n.d.	3.36	PA
**ERNDIM No. 2**	35.62	4277.03	4.30	MMACBL
**ERNDIM No. 3**	91.41	n.d.	2.44	PA
**ERNDIM No. 4**	32.66	24.57	0.21	MMACBL
**ERNDIM No. 5**	117.15	3892.14	8.50	MMACBL
**ERNDIM No. 6**	64.79	0.98	1.32	PA
**ERNDIM No. 7**	39.68	24.55	0.22	MMACBL

3OHPA: 3-hydroxypropionic acid; MMA: methylmalonic acid; MCA: methylcitric acid; n.d.: not detectable; PA: propionic acidemia; MMACBL: methylmalonic acidemia or combined remethylation disorder.

## Discussion

Despite potential benefits of NBS for PA and MMACBL [1, 25], these disorders are currently screened for in only few European countries (Austria, Belgium, Denmark, Hungary, Iceland, Portugal and Spain) [4, 26]. This is in part attributable to relatively high false-positive rates if NBS for these disorders is based solely on C3 results [1]. The relative non-specificity is due to an overlap of C3 concentrations in normal and affected individuals, particularly in prematures [1, 7]. Even if the specificity can be improved if the levels of other biomarkers such as free carnitine, C2, palmitoylcarnitine or methionine are also taken into account in respective ratios with C3 [1, 8], some cases may still be missed reportedly [27]. Thus, there is a need for assays that allow confirmation and differential diagnosis of primary screening results for PA and MMACBL [1]. Urine organic acid analysis by means of gas chromatography–mass spectrometry has been most commonly used, which can simultaneously detect various diagnostic biomarkers including 3OHPA, MMA and MCA (Fig 1) [1]. Other assays have used serum or plasma for the determination of one or two, but not all three, of these relevant biomarkers [28, 29].

In NBS, however, the DBS is the standard sample type due to its advantages, including the ease of sample collection, transportation, storage and stability [30]. The analysis of the same DBS specimen as in the primary screening (second-tier testing) may also reportedly decrease the necessity for recalls, shorten time to diagnosis and improve the cost- and time-effectiveness of screening [9, 31]. Moreover, previous studies highlighted the importance of avoiding unnecessary recalls by reporting significant increases in family anxiety and frequency of hospitalization in subjects with false-positive NBS results [32, 33].

To the best of our knowledge, the LC-MS/MS assay presented here is the first to have multiplexed three relevant biomarkers pathognomonic for PA and MMACBL in DBS (3OHPA, MMA and MCA) in one run, taking into account the separation of the physiological interfering metabolites. Most previous assays using DBS detected MMA and/or MCA, but not in combination with 3OHPA [10–14, 16]. Determination by LC-MS/MS of 3OHPA was reported by two earlier studies which, in contrast with the present method, did not include MCA [15, 17]. To improve quantitation further, we have used a total of three ISs (one for each biomarker) instead of a single IS in these earlier reports [15, 17]. This change may reportedly improve reliability of the assay especially for high analyte levels where, if only one IS is used, suppression of a single IS would affect quantitation of all other analytes [34].

As concerns sample preparation, previous methods generally utilized time-consuming and laborious derivatization steps [10, 11, 13, 14] or liquid-liquid extraction [12]. In our assay, these steps have been eliminated to achieve a very simple and fast (<20 min) sample preparation protocol to minimize errors and increase robustness without any special equipment or further pipetting steps.

Even if our instrument would have been able to perform two-dimensional (2D) chromatography with ultra-high performance, the assay was deliberately developed using one-dimensional (1D) configuration with conventional HPLC settings. This approach, together with simple sample preparation, is expected to improve inter-lab transferability and robustness for an easier inclusion of this assay in the daily routine.

Importantly, due to multiplexing of the biomarkers, our assay allows the evaluation of the individual diagnostic value of 3OHPA, MMA and MCA in the same DBS in the diagnosis and follow-up of PA and MMACBL. As for PA in the present study, MCA was increased all newborn PA samples (*n* = 4) as well as in External Quality Assurance specimens with PA (*n* = 3) and all specimens obtained during therapy (*n* = 21). 3OHPA was also increased in many PA cases. However, in 2 out of 4 untreated newborn PA samples and in 7 out of 21 PA specimens under therapy, only the elevation of MCA, but not 3OHPA, could be detected, in contrast with an earlier study [15]. Of note, PA specimens with elevated MCA but normal 3OHPA originated from PA patients having milder phenotypes. This was also indicated by their lower C3/C2 ratios at time of blood sampling (newborn: range 0.24-0.50, treated: 0.23-0.55) as compared with PA samples having both MCA and 3OHPA increased (newborn: range 0.52-1.94, treated: 0.96-4.31). However, all four newborn PA specimens were subject to long-term storage (range 3.45-8.90 years), leading up to a 95% decrease in the C3 levels, in line with previous reports [23, 24]. It may therefore be hypothesized that pre-analytical issues could have markedly contributed to the lack of detection of 3OHPA in the two NBS samples. It has not been elucidated in the literature whether alterations of C3 are paralleled with those of 3OHPA, MMA and MCA in DBS stored in the long-term. MMA was reported to be stable for at least 1 year in DBS [12], and MCA for at least 3 weeks [11]. In case of PA patients under treatment, our observations might also be attributable to relative metabolic stability under treatment and concomitant therapy with carnitine increasing the renal elimination of the relevant metabolites [1]. Thus, stored newborn and treated PA samples in our study could only be correctly identified if the decision was based on an elevation of MCA. However, these observations may be irrelevant in the analysis of freshly obtained samples. Therefore, in our ongoing prospective study (‟Newborn screening 2020”), we set out to assay emerging fresh specimens from further PA patients with mild or more severe phenotypes to clarify the roles of these factors, as well as the diagnostic value of the biomarkers.

On the other hand, all retrospectively examined newborn (*n* = 10) and External Quality Assurance specimens (*n* = 4) with MMACBL could be clearly distinguished from normal samples based on elevated MMA concentrations; this was accompanied by increased MCA levels in most cases (*n* = 11 out of 14). MMA levels were only moderately elevated and MCA was normal in one untreated newborn with CblB and two with CblC. MMA was also elevated in most MMACBL samples obtained during therapy (*n* = 39 out of 44, including four with normal first-tier results); in 32 out of 39 specimens together with increased MCA levels. In five additional patients under treatment (CblB *n* = 3; CblC *n* = 2), the first-tier and second-tier results were both normal. Thus, in our sample set containing both mild and more severe phenotypes, MMA appears to be superior as diagnostic parameter for the detection of MMACBL in DBS samples from NBS, with MCA being complementary and supporting the decision in most but not all cases.

As concerns maternal vitamin B_12_ deficiency, such patients are expected to be partially identified by the suggested second-tier strategy, as at least severe cases show elevation of C3 and MMA on NBS [35, 36]. Moreover, a clear benefit of early detection and treatment for both the child and the mother in severe maternal vitamin B_12_ deficiency has been reported [37–39]. Thus, in our opinion, the identification of these patients may be regarded as a further advantage of the proposed strategy, in spite of a reported increase in false-positive rates [35–39]. Accordingly, in our prospective study ‟Newborn screening 2020” maternal vitamin B_12_ deficiency is explicitly included as one of the target disorders; the impact on false-positive rates is also under evaluation.

Taken together, our preliminary findings from this retrospective analysis suggest that this assay could reliably identify patients with PA and MMACBL in the present set of NBS samples. Also, most specimens obtained during therapy showed biomarker patterns characteristic for the respective disorder. However, 3OHPA was normal in a relevant number of newborn PA samples–presumably influenced by pre-analytical changes during long-term storage–and also in PA patients under therapy, while MCA could still confirm the presence of PA. As these retrospective results may not be directly applicable for the analysis of freshly obtained samples, the validated assay is being applied prospectively as a second-tier test in our pilot project ‟Newborn screening 2020” at the University Hospital Heidelberg. In addition, that prospective study is expected to provide data on effects of the presented second-tier strategy in NBS on false-positive rates, frequency of recalls and time to diagnosis. More experience in such larger studies representing a wider range of disorders and prospective evaluation of the described method are needed to clarify the relevance of each biomarker in DBS, the reliability of the presented assay to identify patients in the NBS setting and the potential effect on false-positive rates in premature and term newborns.

## Conclusions

In conclusion, an LC-MS/MS assay has been developed for the concurrent determination of the relevant biomarkers pathognomonic for PA and MMACBL in DBS (3OHPA, MMA and MCA), while separating the interfering metabolites lactic acid and succinic acid. Sample preparation is simple and fast (<20 min); no special equipment, derivatization or complicated pipetting steps are required. The assay can use the same DBS specimen as in the primary screening and can therefore be integrated in NBS as a second-tier test to reduce the number of recalls. The method was validated using clinical samples from known patients (newborn specimens before therapy and samples under treatment) and External Quality Assurance specimens. The presented preliminary findings suggest that application of this assay can reliably identify and differentiate patients with PA and MMACBL in NBS samples. The validated assay is currently being evaluated prospectively as second-tier test in a pilot project for the extension of the German NBS panel (‟Newborn screening 2020”) at the NBS Center of the University Hospital Heidelberg.

## Supporting information

S1 Text Preparation of unlabelled standard and internal standard solutions, dried blood spot calibrators and quality controls. (DOC)

S1 Table Extended details of method development. (DOC)
