# Supplementary material for: Simultaneous determination of 3-hydroxypropionic acid, methylmalonic acid and methylcitric acid in dried blood spots: Second-tier LC-MS/MS assay for newborn screening of propionic acidemia, methylmalonic acidemias and combined remethylation disorders
Source: PLoS One. 2017 Sep 15;12(9):e0184897. doi: 10.1371/journal.pone.0184897 (PMC5600371; doi:10.1371/journal.pone.0184897)
Supplement: S1 Text — (DOC) [file pone.0184897.s001.doc]

**S1 Text**

**Manuscript title**

Simultaneous determination of 3‑hydroxypropionic acid, methylmalonic acid and methylcitric acid in dried blood spots: second‑tier LC‑MS/MS assay for newborn screening of propionic acidemia, methylmalonic acidemias and combined remethylation disorders

Péter Monostori^1¶*^, Glynis Klinke^1¶^, Sylvia Richter^1^, Ákos Baráth^2^, Ralph Fingerhut^3^, Matthias R. Baumgartner^3^, Stefan Kölker^1^, Georg F. Hoffmann^1^, Gwendolyn Gramer^1¶^, Jürgen G. Okun^1¶^

^1^ Department of General Pediatrics, Division of Neuropediatrics and Metabolic Medicine, Center for Pediatric and Adolescent Medicine, University Hospital Heidelberg, Heidelberg, Germany

^2^ Department of Pediatrics, University of Szeged, Szeged, Hungary

^3^ Division of Metabolism, Children’s Research Center, University Children’s Hospital Zurich, Zurich, Switzerland

^¶^These authors contributed equally to this work.

*** Corresponding author**

E‑mail: monostoripeter@gmail.com (PM)

**S1 Text: Preparation of unlabelled standard and internal standard (IS) solutions, dried blood spot (DBS) calibrators and Quality Controls (QCs)**

*Preparation of unlabelled standard solutions*

Unlabelled standards 3‑hydroxypropionic acid (3OHPA), methylmalonic acid (MMA) and methylcitric acid (MCA) were dissolved in ultrapure water (18.2 MΩ.cm) separately to obtain 10 mM stock solutions. An intermediate solution mix of unlabelled standards was prepared by mixing stock solutions and diluting with ultrapure water to give final concentrations of 5.0 mM, 0.50 mM and 0.10 mM for 3OHPA, MMA and MCA, respectively. The stock solutions and the intermediate standard solution mix were aliquoted and stored at ‑20 °C.

*Preparation of internal standard solutions*

Deuterated internal standard (IS) d_3_‑lactic acid was dissolved in ultrapure water; d_3_‑MMA and d_3_‑MCA in acetonitrile (ULC‑MS grade) separately to obtain 100 mM, 8.3 mM and 2.5 mM stock solutions, respectively. An intermediate solution mix of ISs was prepared by mixing IS stock solutions and diluting with acetonitrile (ULC‑MS grade) to give final concentrations of 100 μM, 8.3 μM and 2.5 μM for d_3_‑lactic acid, d_3_‑MMA and d_3_‑MCA, respectively. The IS stock solutions and the aliquoted intermediate IS solution mix were stored at ‑20 °C.

*Preparation of DBS calibrators*

A pool of heparinized blood (hematocrit 50%) was spiked with the intermediate solution mix of unlabelled standards to obtain the highest level calibrator (Cal6) at final concentrations of 400 μM, 40 μM and 8.0 μM for 3OHPA, MMA and MCA, respectively. Further calibrators (Cal5 to Cal1) were prepared via 2‑fold serial dilutions of the respective previous calibrator, mixed with equal volumes of the same pooled heparinized blood. Cal0 contained pooled heparinized blood without spiking. Accordingly, analyte levels in DBS calibrators Cal0 to Cal6 were as follows: 0, 12.5, 25, 50, 100, 200, 400 μM for 3OHPA; 0, 1.25, 2.5, 5.0, 10, 20, 40 μM for MMA; and 0, 0.25, 0.50, 1.0, 2.0, 4.0, 8.0 μM for MCA, respectively. All calibrators were mixed and subsequently spotted onto Whatman 903 Neonatal Screening Cards (GE Healthcare Life Sciences, Chicago, IL, USA), dried at room temperature for 24 h and stored at ‑20 °C with silica gel desiccants.

*Preparation of Quality Controls (QCs)*

A pool of heparinized blood (hematocrit 50%) was spiked with appropriate volumes of the intermediate solution mix of unlabelled standards (for QC1) or the 10 mM stock solutions (for QC2 and QC3) to obtain the following analyte levels in QC1, QC2 and QC3: 20, 60, 100 μM for 3OHPA; 2.0, 30, 100 μM for MMA; and 0.40, 1.2, 4.0 μM for MCA, respectively. QCs were spotted, dried and stored similarly to the calibrators.
