# Supplementary material for: Simultaneous determination of 3-hydroxypropionic acid, methylmalonic acid and methylcitric acid in dried blood spots: Second-tier LC-MS/MS assay for newborn screening of propionic acidemia, methylmalonic acidemias and combined remethylation disorders
Source: PLoS One. 2017 Sep 15;12(9):e0184897. doi: 10.1371/journal.pone.0184897 (PMC5600371; doi:10.1371/journal.pone.0184897)
Supplement: S1 Table — (DOC) [file pone.0184897.s002.doc]

**S1 Table**

**Manuscript title**

Simultaneous determination of 3‑hydroxypropionic acid, methylmalonic acid and methylcitric acid in dried blood spots: second‑tier LC‑MS/MS assay for newborn screening of propionic acidemia, methylmalonic acidemias and combined remethylation disorders

Péter Monostori^1¶*^, Glynis Klinke^1¶^, Sylvia Richter^1^, Ákos Baráth^2^, Ralph Fingerhut^3^, Matthias R. Baumgartner^3^, Stefan Kölker^1^, Georg F. Hoffmann^1^, Gwendolyn Gramer^1¶^, Jürgen G. Okun^1¶^

^1^ Department of General Pediatrics, Division of Neuropediatrics and Metabolic Medicine, Center for Pediatric and Adolescent Medicine, University Hospital Heidelberg, Heidelberg, Germany

^2^ Department of Pediatrics, University of Szeged, Szeged, Hungary

^3^ Division of Metabolism, Children’s Research Center, University Children’s Hospital Zurich, Zurich, Switzerland

^¶^These authors contributed equally to this work.

*** Corresponding author**

E‑mail: monostoripeter@gmail.com (PM)

**S1 Table: Extended details of method development.**

This table is intended to share our experience obtained during method development, as well as reasons why a given configuration was selected for subsequent method validation.

| **Assay parameter** | **Configurations tested** | **Notes** |
| --- | --- | --- |
| **Extraction solvent** | ACN/water 20/80 (v/v)  ACN/water 40/60 (v/v)  ACN/water 60/40 (v/v)  ACN/MeOH/water 20/20/60 (v/v)  ACN/MeOH/water 20/40/40 (v/v)  ACN/water 20/80 (v/v) +0.4% formic acid  ACN/water 20/80 (v/v) +0.1% acetic acid | Peak intensities and signal‑to‑noise ratios were the highest using ACN/MeOH/water 20/40/40 (v/v). |
| **Sample preparation technique** | Underivatized  Derivatized with 3.0N hydrochloric acid in n‑butanol | Only the underivatized sample preparation allowed detection of 3OHPA. It was also simpler and faster, with higher peak intensities for MCA (peak intensities for MMA were lower than with derivatization). |
| **Column** | Waters Acquity BEH Amide 150x2.1 mm, 1.7 μm  Waters Acquity BEH C18 100x2.1 mm, 1.7 μm  Waters Atlantis HILIC Silica 50x2.1 mm, 3 μm  Phenomenex Luna Omega Polar C18 100x2.1 mm, 1.6 μm  Phenomenex Gemini C6‑Phenyl 150x2.0 mm, 3 μm | Columns were selected for testing on the basis of literature data and analyte properties. The Phenomenex Gemini C6‑Phenyl 150x2.0 mm, 3 μm column provided best resolution, peak shape and signal intensity among the tested columns. |
| **Eluents** | A: Water +0.1% formic acid  A: Water +0.4% formic acid  A: Water +0.1% acetic acid  B: ACN/MeOH 50/50 (v/v)  B: ACN/MeOH 50/50 (v/v) +0.4% formic acid  B: ACN/water 80/20 (v/v) +0.1% formic acid  B: ACN +0.1% formic acid | Resolution and peak shape was most optimal with water +0.4% formic acid as Eluent A and ACN/MeOH 50/50 (v/v) as Eluent B. |
| **LC method type** | Isocratic: 85% Eluent A (water +0.4% formic acid) and 15% Eluent B (ACN/MeOH 50/50 (v/v)); flow rate 200 μl/min.  Gradient: Eluents A and B and flow rate are the same as above, but %B is changed as follows: 0.0‑1.5 min: 10%; 1.5‑2.5 min: 10% to 30%; 2.5‑4.0 min: 30%; 4.0‑4.1 min: 30% to 10%; 4.1‑10.0 min: 10%. | Using the gradient LC method, the LOD and LLOQ values for 3OHPA and MMA were lower:  Isocratic LOD: 40 μM, 3.5 μM, 0.05 μM.  Isocratic LLOQ: 50 μM, 5.0 μM, 0.07 μM.  Gradient LOD: 15 μM, 1.5 μM, 0.05 μM.  Gradient LLOQ: 20 μM, 2.5 μM, 0.07 μM.  (Data are provided in the order 3OHPA, MMA and MCA, respectively.)  For analyte levels above the LLOQ, interday and intraday precisions, variations between injections and recoveries were similar for both the gradient and isocratic LC methods. |

Underlined configurations were selected for subsequent method validation.

ACN: acetonitrile; MeOH: methanol; 3OHPA: 3‑hydroxypropionic acid; MMA: methylmalonic acid; MCA: methylcitric acid; LC: liquid chromatography; LOD: limit of detection; LLOQ: lower limit of quantitation.
